# First detection of two cycloviruses in cormorant fecal samples in China by high-throughput sequencing technology

**DOI:** 10.3389/fvets.2025.1677378

**Published:** 2025-09-16

**Authors:** Yifei Pei, Shiyu Cai, Yue Xue, Yutong Fu, Jingyue Zhang, Quan Shen, Likai Ji, Ping Wu, Hua Wang, Yan Wang, Wen Zhang, Shixing Yang

**Affiliations:** ^1^School of Medicine, Jiangsu University, Zhenjiang, Jiangsu, China; ^2^Department of Rehabilitation, Danyang Hospital of Traditional Chinese Medicine, Danyang Jiangsu, China; ^3^Department of Biology, Shenzhen MSU-BIT University, Shenzhen, Guangdong, China

**Keywords:** viral metagenomics, circovirus, sequence alignment, phylogenetic analysis, genomic structure

## Abstract

**Introduction:**

The Great Cormorant (*Phalacrocorax carbo*) is widely distributed across China. As an apex predator in aquatic ecosystems, it plays a tripartite ecological role: acting as a natural host, transmission vector, and indicator species for viruses. Current research confirms that cormorants carry diverse viral pathogens from the families including *Flaviviridae, Orthomyxoviridae, Paramyxoviridae*, and *Polyomaviridae*. Significant knowledge gaps persist regarding their virome diversity.

**Methods:**

In this study,46 cormorant fecal swab samples were collected at Xiamen Garden Expo Park, and viralmetagenomics method was conducted to identify two Cycloviruses.

**Results:**

This study identified two novel cycloviruses, Corcyclo-1 (1,856 bp) and Corcyclo-2 (1,831 bp), from cormorant fecal samples using viral metagenomics. Genomic analyses revealed hallmark features of the genus Cyclovirus, including inversely oriented open reading frames (ORFs) encoding the capsid protein (Cap) and replication-associated protein (Rep), as well as a conserved stem-loop sequence TAATACTAT. The Rep gene of Corcyclo-1 contained a 166-bp intron and shared >96.9% amino acid identity with human-, wild boar-, and chicken-derived cyclovirus strains (HaCV-8) from Vietnam and Madagascar, classifying it as a novel strain of HaCV-8. In contrast, Corcyclo-2 harbored a 98-bp intron in its Rep gene and clustered with unclassified cyclovirus strains from bats and mongooses in China and Saint Kitts and Nevis (>97.4% identity), constituting a putative new species. Phylogenetic and pairwise sequence analyses further supported their taxonomic positions. Epidemiological screening demonstrated a high prevalence of Corcyclo-1 (82.6%, 38/46) and Corcyclo-2 (32.6%, 15/46) in cormorant feces. Cross-species surveillance detected Corcyclo-2 in chickens (25.8%, 16/62) and ducks (11.7%, 9/77), whereas Corcyclo-1 was absent in these hosts.

**Conclusion:**

This study represents the first report of cormorant-associated cycloviruses, highlighting their potential for cross-species transmission and providing new insights into the ecological diversity and evolutionary mechanisms of cyclovirus.

## Introduction

The Great Cormorant is a widely distributed waterbird across global coastal and inland aquatic ecosystems (1, 2). According to monitoring data from BirdLife International, the global Great Cormorant population is estimated at 1.2 to 2.4 million individuals. However, overfishing in some regions has caused a sharp decline in their food resources. In China, coastal pollution in traditional wintering grounds such as Bohai Bay has resulted in local population declines exceeding 30%. Mass infections by cestode parasites can significantly increase juvenile mortality rates (3). Despite the Great Cormorant's crucial ecological role in marine food chains, systematic research on its virome remains lacking.

The family *Circoviridae* comprises covalently closed circular single-stranded DNA (ssDNA) viruses, representing the smallest known animal-infecting viral group (4, 5). They are classified within two genera, *Circovirus* and *Cyclovirus* (4, 6). *Circoviruses* exhibit cross-species transmission capabilities among Anseriformes (waterfowl) and various other aquatic birds, causing clinical manifestations including immunosuppression, developmental disorders, and digestive disturbances (7–9). In poultry, chickens, turkeys, and guinea fowl are highly susceptible to *Avian circovirus*. Primarily transmitted via the fecal-oral route, this virus causes characteristic pathological lesions such as bursal atrophy and feather dystrophy (10). Wild bird surveillance indicates *Circoviridae* family members exhibit a 34% positive detection rate in Columbiformes (e.g., the Hill Pigeon, Columba rupestris), with infected individuals experiencing persistent weight loss and diminished flight capacity (11, 12). Although circovirus prevalence in Anseriformes remains unclear, their broad host adaptability suggests a potential zoonotic transmission risk to Bar-headed Goose (Anser indicus) populations (8). Moreover, a previous study reported that a novel human circovirus, HuCV2, was discovered in intravenous drug users in China, showing the highest homology with porcine circovirus type 3 (PCV3) (13). This has heightened concerns about the cross-species transmission of circoviruses.

The genus *Cyclovirus*, classified within the family *Circoviridae*, comprises small, non-enveloped viruses with covalently closed circular single-stranded DNA (ssDNA) genomes (1.7–2.1 kb) (4). First identified in 2010, cycloviruses share structural similarities with circoviruses, including bidirectional open reading frames (ORFs) encoding the capsid (Cap) and replication-associated (Rep) proteins, as well as a conserved stem-loop motif (TAATACTAT) near the origin of replication (14). However, cycloviruses exhibit distinct genomic features such as frequent introns in the Rep gene and lower overall nucleotide identity with circoviruses (<70%), justifying their classification as a separate genus (15).

Cycloviruses have been detected in diverse hosts, including humans, livestock, wildlife (e.g., bats, rodents, and birds), and environmental samples, suggesting broad host tropism and potential zoonotic transmission (16, 17). Notably, human-associated cycloviruses (e.g., HaCV-8) have been linked to neurological and respiratory infections, though their pathogenicity remains poorly understood (14). In animals, cyclovirus infections are often subclinical, but their role in immunosuppression or coinfections warrants further investigation.

Despite their ecological prevalence, cyclovirus research lags behind circoviruses due to challenges in culturing and the lack of standardized detection protocols. Wild birds, particularly aquatic species, are underrepresented in cyclovirus surveillance, leaving gaps in understanding their evolutionary dynamics and cross-species transmission risks. This study addresses this gap by characterizing novel cycloviruses in Great Cormorants, a keystone species in aquatic ecosystems.

Viral metagenomics technology has been successfully applied for pathogen screening in various waterfowl species, offering a viable approach to study viral diversity and potential health risks in this species (18, 19). This study employed viral metagenomics to analyze 46 fecal samples from wild Great Cormorants collected in Xiamen, Fujian Province during 2021. This study reports the first full-genome characterization of a great cormorant-associated circovirus, revealing molecular evidence that wetland waterbirds harbor viral genes homologous to human/mammalian viruses. The complete viral genome sequences obtained provide essential data for establishing molecular detection standards targeting the Cap gene for avian circoviruses in southeastern coastal China. This has significance for monitoring viral diseases in Great Cormorants and assessing zoonotic transmission risk.

## Materials and methods

### Sample collection and preparation

**Figure 1:**
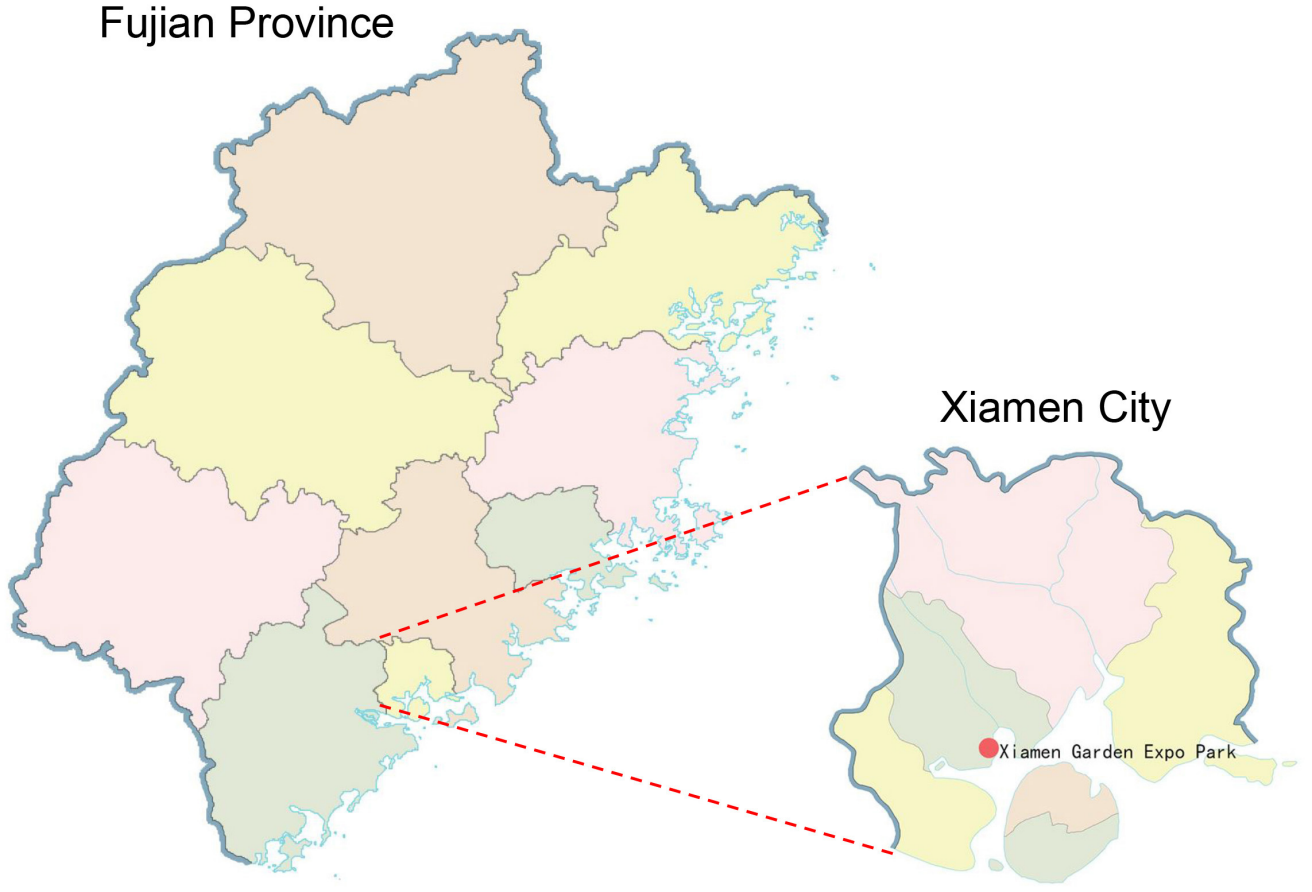
The geographical position of the sample site (red dot) in Xiamen, Fujian, China.

In 2021, 46 cormorant fecal swab samples were collected at Xiamen Garden Expo Park, with species identification confirmed by ornithologists during collection (Figure 1). The swabs were soaked in 1 mL of Dulbecco's phosphate-buffered saline (DPBS) and vortexed for 10 min. Following centrifugation, the supernatant was moved to a 1.5 mL tube and kept at −80 °C for later analysis. The research was authorized by Jiangsu University's Ethics Committee (Approval No. 2018UJS18023), and sampling followed China's Wildlife Protection Law. The following experimental procedures were conducted following the methods described by Yijie Sun et al. (8).

### Viral nucleic acid extraction and library construction

A pool of 9–10 swab extracts was used to construct each library, generating five libraries (swab39, swab40, swab41, swab42, and swab43). Each library used 500 μL of swab suspension (50–55.6 μL supernatant per sample), which was filtered using a 0.45-μm filter (Merck Millipore, USA) to exclude bacteria and eukaryotic cells. The filtrate underwent nuclease treatment (Turbo DNase from Thermo Fisher Scientific, USA, BaselineZero DNase from the US epicenter, Benzoase nuclease from Novagen Corporation, USA, and RNase A from Thermo Fisher Scientific) at 37 °C for 90 min to digest exposed nucleic acids. The QIAamp MinElute Virus Spin Kit (Qiagen, Germany) was employed for viral nucleic acid extraction, followed by concentration measurement with a Qubit 4 fluorometer (Invitrogen, USA). Purified nucleic acids were stored at −80 °C.

The combined viral nucleic acids (DNA/RNA) were reverse transcribed using SuperScript III Reverse Transcriptase (Invitrogen, USA) with 100 pmol random hexamers. The RT protocol included: 25 °C for 10 min, 50 °C for 60 min, 85 °C for 5 min, and 95 °C for 2 min. Products were rapidly cooled and kept on ice for 2 min. cDNA synthesis was completed with Klenow fragment (NEB, USA) at 37 °C for 60 min and 75 °C for 20 min.

The Nextera XT DNA Sample Preparation Kit (Illumina, USA) was used for library preparation following standard protocols, including adapter ligation and 15 cycles of limited amplification. Personalbio conducted sequencing on the Illumina NovaSeq platform, generating 250-bp paired-end reads with dual-indexing.

### Bioinformatic processing pipeline

The NovaSeq platform produced 250-bp paired-end reads, which were demultiplexed using Illumina's software and analyzed through a custom bioinformatics pipeline running on a 32-node Linux cluster. Adapters were removed using VecScreen with default settings. Sequence ends with Phred scores below 10 were removed. Bowtie2 v2.2.4 aligned reads against the BLAST NT database to eliminate bacterial sequences (20). Remaining reads were reconstructed with SOAPdenovo2 (k-mer = 63, default settings) (21). Contigs and singlets were matched to a custom viral proteome database through BLASTx analysis (E-value cutoff <10^−5^). This viral database was curated from NCBI's viral reference proteome FASTA files (https://ftp.ncbi.nih.gov/refseq/release/viral/) using taxonomy annotation from the viral kingdom. Potential viral sequences underwent additional screening against an NVNR protein database to exclude false identifications.

### Sequence alignment and ORF prediction

Pairwise alignment of viral amino acid sequences was performed using SDTv1.2 with MUSCLE parameters and Geneious 11.1.2 with MUSCLE alignment. ORFs were identified using Geneious 11.1.2 and NCBI ORF Finder.

### Phylogenetic analysis

Phylogenetic analysis examined evolutionary relationships using amino acid sequences of circoviruses identified in this study, along with top BLASTx matches and representative viral strains (22). Sequence alignments were performed with MUSCLE in MEGA X. MrBayes v3.2.7 generated trees with mixed amino acid models (“prset aamodelpr = mixed”). The analysis ran for 10 million generations (sampling every 50), discarding initial 25% as burn-in. Convergence was achieved at split frequency SD < 0.01, with bootstrap values assigned to nodes.

### Primer development and cyclovirus detection by PCR

Two sets of primers were designed based on the Cap gene of Corcyclo-1 and Corcyclo-2. Using these primers, we performed PCR on DNA extracted from fecal samples of cormorants, chickens, and ducks. PCR reactions (25 μL total volume) contained 3 μL DNA template, 10 μL HotStarTaq Master Mix, 2 μL each primer (20 pmol), and ddH_2_O. Controls included positive and negative samples (extraction/water controls). Cycling parameters: 95 °C for 2 min; 30 cycles of 94 °C (20 s), 55 °C (30 s), 72 °C (30 s); final extension at 72 °C for 5 min. Amplicons were bidirectionally sequenced ≥2 times via Sanger sequencing (Sangon Biotech, China).

### Nucleotide sequence accession number

The complete viral genome sequences identified in this study were deposited in GenBank under the accession numbers VP731248 and VP731249.

## Results

### Viral metagenomic overview

**Figure 2:**
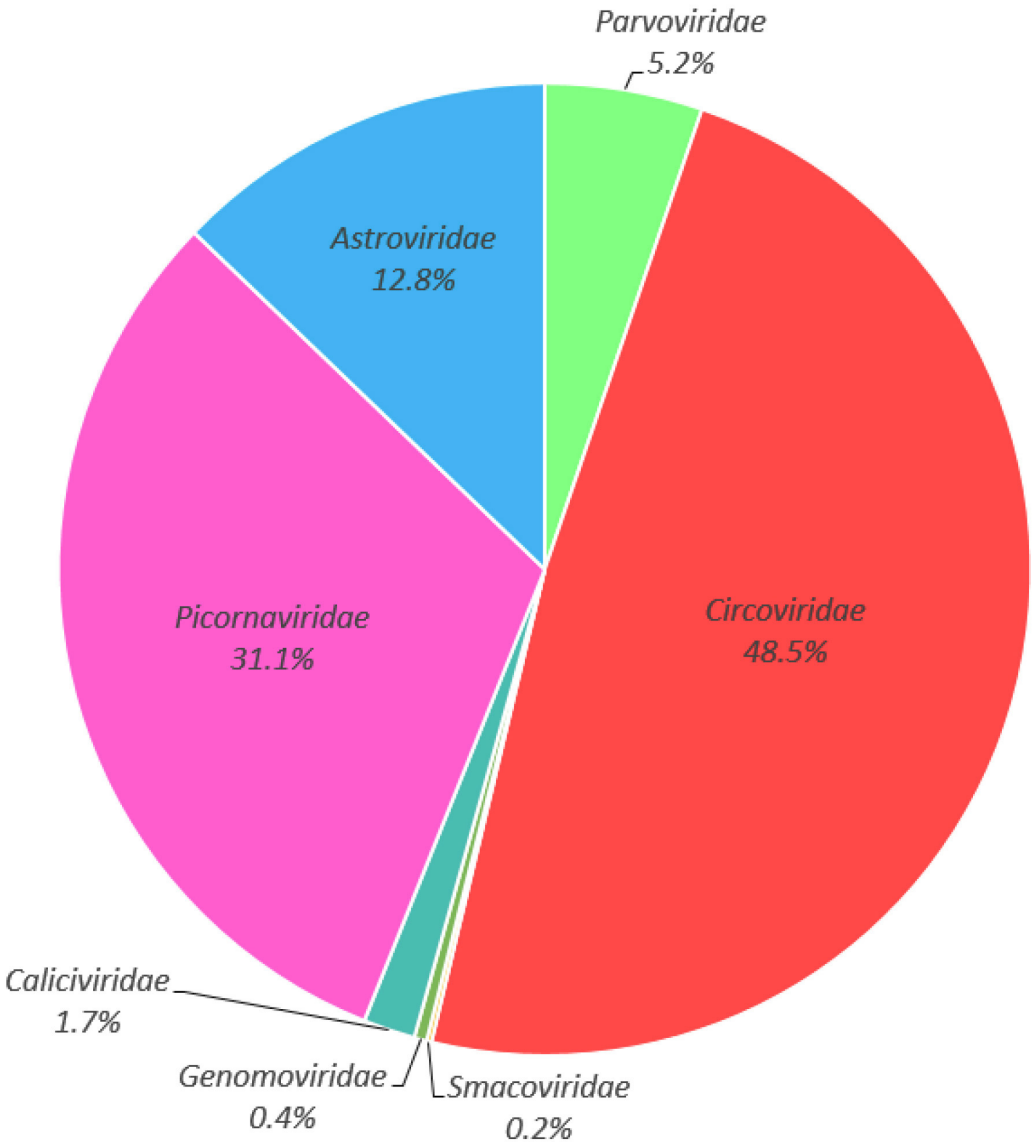
Taxonomic analysis of the fecal virome detected in cormorants at the family level.

Illumina sequencing generated 13,877,028 raw reads across five libraries (Supplementary Table S1). After bioinformatics analysis, a total of 568,343 sequences reads matching viral proteins, accounting for 4.1% of the total number of raw reads. The majority of the eukaryotic viruses identifies in this study including the families of *Circoviridae* (48.5% of the total analyzed virus reads), *Picornaviridae* (31.1%), *Astroviridae* (12.8%), *Parvoviridae* (5.2%), *Caliciviridae* (1.7%), *Genomoviridae* (0.4%), and *Smacoviridae* (0.2%) (Figure 2, Supplementary Table S2). Considering the potential pathogenic role, and the fact that no circovirus has been detected in cormorants to date, we chose the circoviruses for future comprehensive study.

### The genomic analysis of two novel cycloviruses

**Figure 3:**
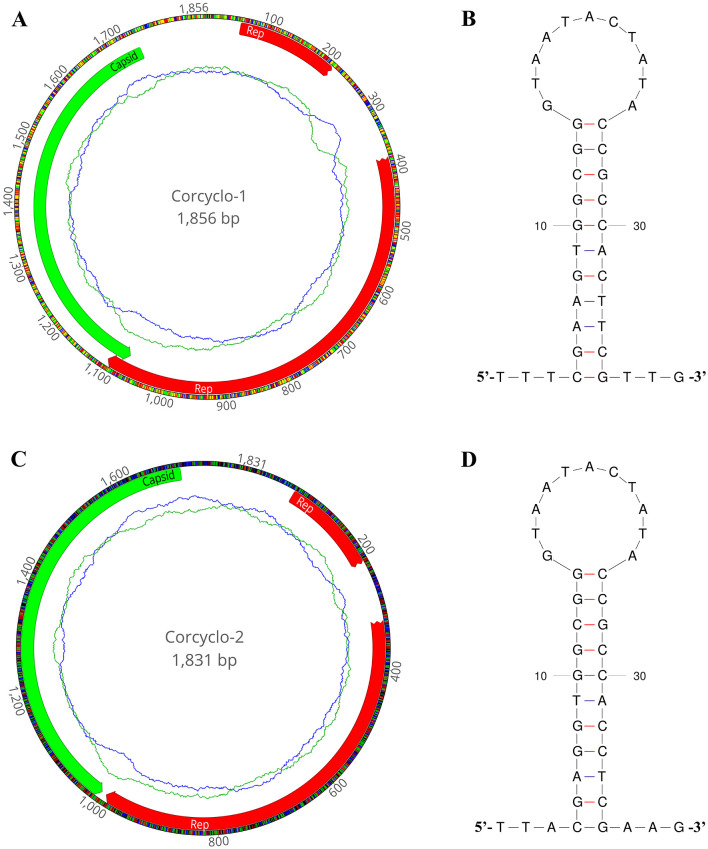
The genomic organization and stem-loop structures of cycloviruses identified in cormorants. **(A)** The genomic organization of Corcyclo-1 identified in cormorants; **(B)** the stem-loop structure of Corcyclo-1 identified in cormorants; **(C)** the genomic organization of Corcyclo-2 identified in cormorants; **(D)** the stem-loop structure of Corcyclo-2 identified in cormorants.

In this study, *De novo* assemble yielded two complete cyclovirus genomes: Corcyclo-1 (1,856 bp) and Corcyclo-2 (1,831 bp). Both genomes exhibited hallmark features of the genus *Cyclovirus* including two inversely oriented open reading frames encoding the Cap protein and Rep protein (23). The GC contents of Corcyclo-1 and Corcyclo-2 are 47.2 % and 47.5 %, respectively. For Corcyclo-1, ORF1 is 669 bp in length encoding a 222-aa Cap protein, while ORF2 is 879 bp in length encoding a 292-aa Rep protein. For Corcyclo-2, Cap protein encoded by ORF1 is 232 aa in length, while Rep protein is 277 aa (Figures 3A, C). The Rep genes of both cycloviruses were predicted to be interrupted by a 166-bp or 98-bp intron with a typical splice donor (GT) and splice acceptor site (AG) respectively. The conserved motifs of cyclovirus (WWDGY, DDFYGW, DRYP, CSK) were presented in the Rep proteins of both Corcyclo-1 and Corcyclo-2. The typical nonamer sequence of the stem loop structure (TAATACTAT) was conserved in the genomes of Corcyclo-1 and Corcyclo-2 (Figures 3B, D). Despite structural similarities, Corcyclo-2 lacked ORF overlap due to a premature termination codon in its Rep gene.

### Phylogenetic analysis

**Figure 4:**
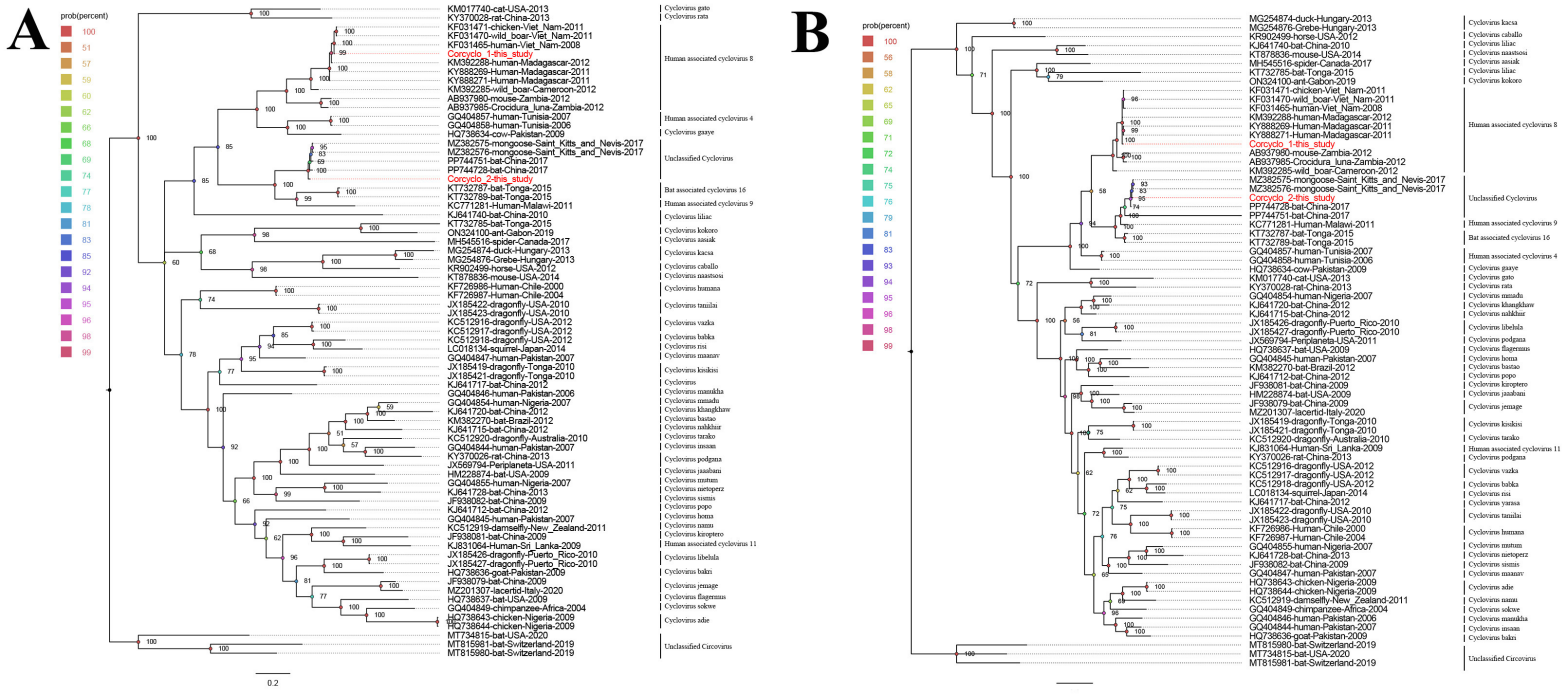
Phylogenetic analysis of cycloviruses identified in cormorants. **(A)** phylogenetic tree constructed based on Cap proteins; **(B)** phylogenetic tree constructed based on Rep proteins. Viruses identified in this study are marked with red.

Two phylogenetic trees were constructed using Cap and Rep protein sequences from Corcyclo-1 and Corcyclo-2, and reference strains from the Cyclovirus species (Figures 4A, B). For Corcyclo-1, Cap phylogeny clustered it with other cyclovirus strains isolated from cerebrospinal fluid and feces samples from human (GenBank no. KF031465 and KM392288), wild boar (GenBank no. KF031470), and chicken (GenBank no. KF031471). Both of them belong to Human associated cyclovirus 8 (HaCV-8). Rep phylogeny further supported this classification, suggesting cross-species transmission potential. For Corcyclo-2, both Cap and Rep phylogenies grouped it with unclassified cyclovirus strains from feces samples of bat and mongoose.

### Pairwise alignment of amino acid sequences and nucleotide sequences

**Figure 5:**
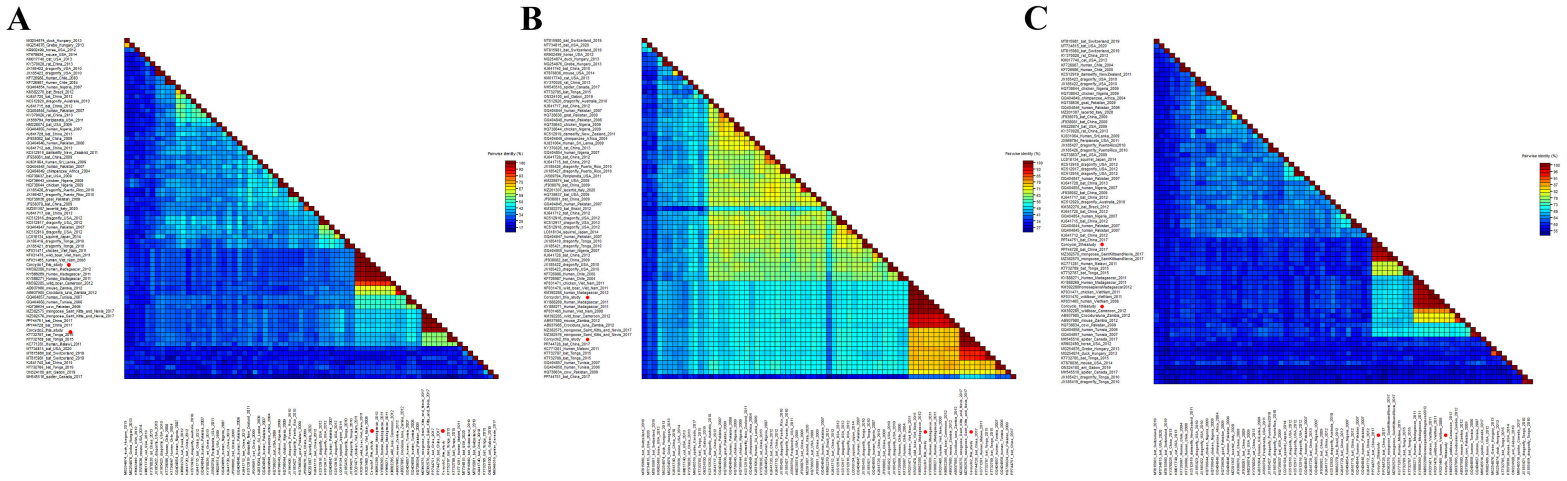
Pairwise comparison of nucleotide and amino acid sequences of cycloviruses identified in cormorants with representative strains of different species and genera in the *Circoviridae* family. **(A)** pairwise comparison based on Cap proteins; **(B)** pairwise comparison based on Rep proteins; **(C)** pairwise comparison based on nucleotide sequences of full genomes.

In order to determine the classification of these cycloviruses detected in this study, the viral encoded proteins and whole genome sequences were compared with the corresponding sequences of known cycloviruses. For Corcyclo-1, Cap protein alignment revealed >96.9% amino acid identity with cyclovirus strains from humans, wild boars, and chickens in Vietnam and Madagascar, and <92% identity with other representative strains (Figure 5A). Rep alignment showed >97.2% identity with strains from humans, wild boars, and chickens in Vietnam and Madagascar, but <92% identity with other strains (Figure 5B). Whole-genome alignment exhibited >96.9% nucleotide identity with strains from humans, wild boars, and chickens in Vietnam and Madagascar, and <86.3% identity with others strains (Figure 5C). For Corcyclo-2, Cap alignment indicated >97.4% amino acid identity with unclassified cyclovirus strains from bat and mongoose in China and Saint Kitts and Nevis, but <53.5% identity with other representatives (Figures 5A, B). Rep alignment showed >99.3% identity with unclassified cyclovirus strains from bat and mongoose in China and Saint Kitts and Nevis, while <91.4% identity with other strains. Whole-genome alignment exhibited >96.9% nucleotide identity with Cyclovirus strains from bat and mongoose in China and Saint Kitts and Nevis, and <88.9% with others strains (Figure 5C). Viral taxonomy standards require ≤ 80% whole-genome nucleotide similarity and consistent topological positioning of the replication origin (ori) relative to Rep/Cap coding regions for species classification within a genus (24). Based on this classification standard, Corcyclo-1 should be classified as Human associated cyclovirus 8 of the *Hepatovirus* genus, while Corcyclo-2 is classified as an unclassified cyclovirus.

### Cyclovirus prevalence estimation

To investigate the prevalence of Corcyclo-1 and Corcyclo-2, viral DNA genomes were extracted from all 46 fecal samples. Two sets of primers, designed based on the Cap gene of Corcyclo-1 and Corcyclo-2, were respectively used to perform PCR screening (Supplementary Table S3). The results indicated that 38 samples were positive for Corcyclo-1 (38/46, 82.6% positive rate), while only 15 samples were positive for Corcyclo-2 (15/46, 32.6% positive rate) (Supplementary Figures S1, S2). Since both cycloviruses have previously been detected in other animal species, we investigated their cross-species prevalence by screening 62 chicken and 77 duck fecal samples from surrounding areas using the above primers. Results showed that Corcyclo-2 was detected in both sample types, with positive rates of 25.8% (16/62) in chickens and 11.7% (9/77) in ducks (Supplementary Figures S3, S4), in contrast, Corcyclo-1 was not detected in fecal samples from chickens and ducks.

## Discussion

Birds harbor diverse viromes, and the viral diversity in healthy individuals suggests their role as natural viral reservoirs (18). The Great Cormorant is protected by China's National Forestry and Grassland Administration and widely distributed. Previous studies identified multiple pathogenic viruses infecting this species: Nazerian et al. (25) first isolated a novel herpesvirus from diseased chick blood; Bányai et al. (25) discovered a novel gammapolyomavirus in deceased individuals at Budapest Zoo, Hungary; Varsani et al. (26) identified multiple viruses (including Cressdnavirus, Microvirus, Gyrovirus, and Caudovirus) in fecal samples of Double-crested Cormorants in the United States (27). Currently, systematic surveillance data on viral carriage or infection in Chinese cormorant populations are absent.

The family *Circoviridae* currently comprises approximately 155 species classified into two genera (*Circovirus* and *Cyclovirus*) (6). Breakthroughs in PCR and metagenomic sequencing have significantly expanded our understanding of their diversity (28, 29). Current data reveal that members of the genus Circovirus are widely distributed in human clinical samples (positivity rate 2.9%−7.2%), livestock hosts (15%−34% positivity in domestic pigs), and aquatic environments (89% positivity in wastewater treatment plants), demonstrating remarkable environmental adaptability (30–33).

This study identified and characterized two novel cycloviruses in cormorant fecal samples. The discovery of Corcyclo-1 and Corcyclo-2 significantly expands knowledge regarding the genetic diversity and host range of *Circoviridae*. Full genome sequencing and phylogenetic analysis indicate that Corcyclo-1 belongs to HaCV-8. In this study, HaCV-8 was found to have a high prevalence in cormorants, but it was not detected in the fecal samples of chickens and ducks from the same area. Further epidemiological investigations are needed to determine its pathogenicity in cormorants and assess its potential for transmission to humans. In contrast, evolutionary analysis and sequence comparison showed that Corcyclo-2 discovered in this study is closely related to the unclassified cyclovirus in the feces of wild bats in China and the cyclovirus in the feces of mongooses in Saint Kitts and Nevis, and it is classified as an unknown novel cyclovirus. Epidemiological surveys showed that Corcyclo-2 was also detected in the fecal samples of chickens and ducks from the same area, indicating that it can infect multiple species. However, whether it is pathogenic to the infected animals needs further detailed research. Since Corcyclo-2 was detected in cormorants, chickens, and ducks in this study, and genetically similar strains have previously been identified in bats, we hypothesize that the virus is capable of cross-species transmission. Transmission is likely mediated by the fecal–oral route, and contact transmission cannot be excluded. Therefore, continuous surveillance of Corcyclo-2 infection in wild birds and domestic poultry is essential for the early detection and control of its potential pathogenic threat to both animal and public health.

To date, novel cyclovirus-like viruses have been isolated from diverse species, indicating broad host infectivity. Although no significant avian clinical signs were observed during sampling, undetected clinical disease due to insufficient sampling cannot be excluded. Consequently, further experimental and epidemiological studies are needed to elucidate their pathogenicity and host range. While this study advances understanding of cormorant-associated viruses, limitations remain. First, reliance solely on fecal samples precludes comprehensive viral distribution assessment, as these viruses may inhabit other tissues or biological fluids. Second, despite discovering Corcyclo-1 and Corcyclo-2, functional validation of their pathogenicity and transmission modes under natural conditions is lacking. Finally, sampling spatiotemporal constraints may limit representation of population-wide viral carriage, especially given potential geographical and seasonal influences on viral diversity and abundance.

## Conclusion

This study reports the first identification of two novel viruses, Corcyclo-1 and Corcyclo-2, substantially enhancing our knowledge of viral diversity carried by wild cormorants. Both Corcyclo-1 and Corcyclo-2 are members of the family *Circoviridae*. Corcyclo-1 exhibits significant homology and structural similarity to Human associated cyclovirus 8 (HaCV-8), a virus whose nucleic acid has been detected in human samples. However, the clinical significance and pathogenicity of HaCV-8 in humans remain entirely unknown. The high prevalence of this virus in cormorants warrants further investigation to determine its true host range and to assess the potential risk of zoonotic transmission. Corcyclo-2 is closely related to several unclassified cycloviruses, forming an independent branch and is designated as an unclassified cyclovirus. Epidemiological investigations showed that Corcyclo-2 could also infect chickens and ducks in the same region, while Corcyclo-1 was not detected in fecal samples from chickens and ducks. These findings contribute to understanding the genetic diversity and host range of these viruses. Future research should better expand the cormorant sample size and characterize the epidemiology, pathogenicity, and potential for cross-species transmission of these newly discovered viruses.

## Data Availability

The complete viral genome sequences identified in this study were deposited in GenBank under the accession numbers PV731248 and PV731249.

## Supplementary material

Supplementary Table S1 The summary of library information of cormorant.

Supplementary Table S2 The sequence numbers of eukaryotic viruses in family level in different libraries.

Supplementary Table S3 The summary of primer information of *Corcyclo-1* and *Corcyclo-2*.

Supplementary Figure S1 PCR screening results for *Corcyclo-1* in cormorant fecal samples.

Supplementary Figure S2 PCR screening results for *Corcyclo-2* in cormorant fecal samples.

Supplementary Figure S3 PCR screening results for *Corcyclo-2* in chicken fecal samples.

Supplementary Figure S4 PCR screening results for *Corcyclo-2* in duck fecal samples.
