# Supplementary material for: First detection of two cycloviruses in cormorant fecal samples in China by high-throughput sequencing technology
Source: Front Vet Sci. 2025 Sep 16;12:1677378. doi: 10.3389/fvets.2025.1677378 (PMC12481609; doi:10.3389/fvets.2025.1677378)
Supplement: Supplementary Table S1 — The summary of library information of cormorant. [file Table_1.docx]

Table S1. The summary of library information of cormorant

| Library ID | Sample type | No. of sample | Healthy status | Total no. of raw reads | No. of viral reads with E value<10^-5^ |
| --- | --- | --- | --- | --- | --- |
| swab39 | Feces | 9 | healthy | 2,205,606 | 24,038 |
| swab40 | Feces | 9 | healthy | 4,609,598 | 55,669 |
| swab41 | Feces | 9 | healthy | 2,094,080 | 322,554 |
| swab42 | Feces | 9 | healthy | 3,466,014 | 156,953 |
| swab43 | Feces | 10 | healthy | 1,501,730 | 9,129 |
