# Supplementary material for: First detection of two cycloviruses in cormorant fecal samples in China by high-throughput sequencing technology
Source: Front Vet Sci. 2025 Sep 16;12:1677378. doi: 10.3389/fvets.2025.1677378 (PMC12481609; doi:10.3389/fvets.2025.1677378)
Supplement: Supplementary Table S2 — The sequence numbers of eukaryotic viruses in family level in different libraries. [file Table_2.docx]

**Table S2.** The sequence numbers of eukaryotic viruses in family level in different libraries.

| Virus Families | No. of viral reads with E value<10^-5^ | | | | |
| --- | --- | --- | --- | --- | --- |
|  | swab39 | swab40 | swab41 | swab42 | swab43 |
| *Parvoviridae* | 48 | 339 | 0 | 0 | 296 |
| *Circoviridae* | 17 | 109 | 3,846 | 2,324 | 22 |
| *Smacoviridae* | 22 | 0 | 0 | 0 | 0 |
| *Genomoviridae* | 0 | 44 | 0 | 0 | 0 |
| *Caliciviridae* | 0 | 218 | 0 | 0 | 7 |
| *Picornaviridae* | 78 | 329 | 3,033 | 206 | 409 |
| *Astroviridae* | 0 | 691 | 573 | 406 | 0 |
