# Supplementary material for: First detection of two cycloviruses in cormorant fecal samples in China by high-throughput sequencing technology
Source: Front Vet Sci. 2025 Sep 16;12:1677378. doi: 10.3389/fvets.2025.1677378 (PMC12481609; doi:10.3389/fvets.2025.1677378)
Supplement: Supplementary Table S3 — The summary of primer information of Corcyclo-1 and Corcyclo-2. [file Table_3.docx]

Table S3. The summary of primer information of *Corcyclo-1* and *Corcyclo-2*.

| Strains name | Primer name | Primer sequences | Amplicon length |
| --- | --- | --- | --- |
| *Corcyclo-1* | *Corcyclo-1-F1* | 5’-TTGATCTTGCGACCAGTGGT-3’ | 175 bp |
|  | *Corcyclo-1-R1* | 5’-TGGCACGACGACTACTTTCC-3’ |  |
| *Corcyclo-2* | *Corcyclo-2-F2* | 5’-ACATGCTGCTGAGAACCCAA-3’ | 154 bp |
|  | *Corcyclo-2-R2* | 5’-CTCCGTTGTTCGTGATTGCC-3’ |  |
