# Supplementary figures and images for: First detection of two cycloviruses in cormorant fecal samples in China by high-throughput sequencing technology
Source: Front Vet Sci. 2025 Sep 16;12:1677378. doi: 10.3389/fvets.2025.1677378 (PMC12481609; doi:10.3389/fvets.2025.1677378)

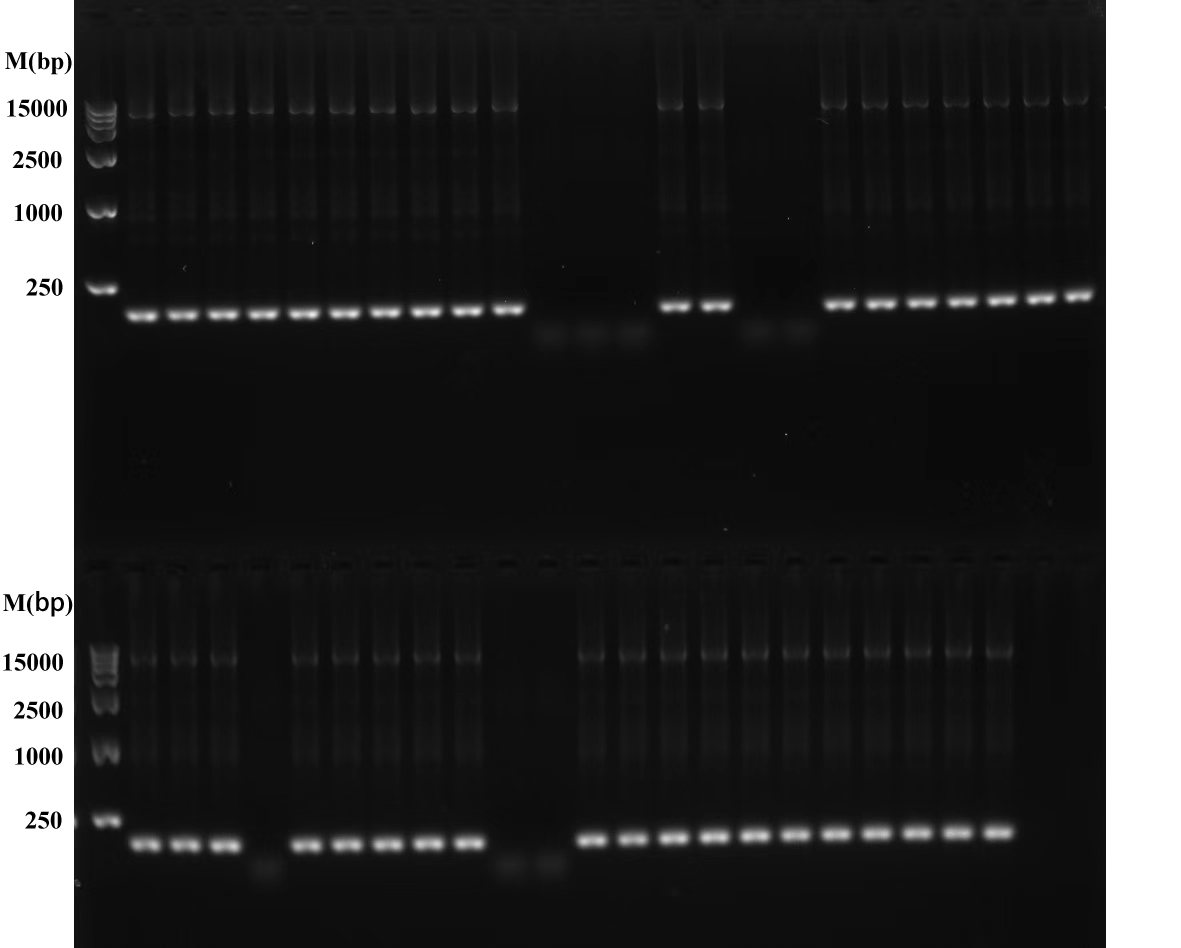

Supplement: Supplementary Figure S1 — PCR screening results for Corcyclo-1 in cormorant fecal samples. [file Image_1.jpeg]

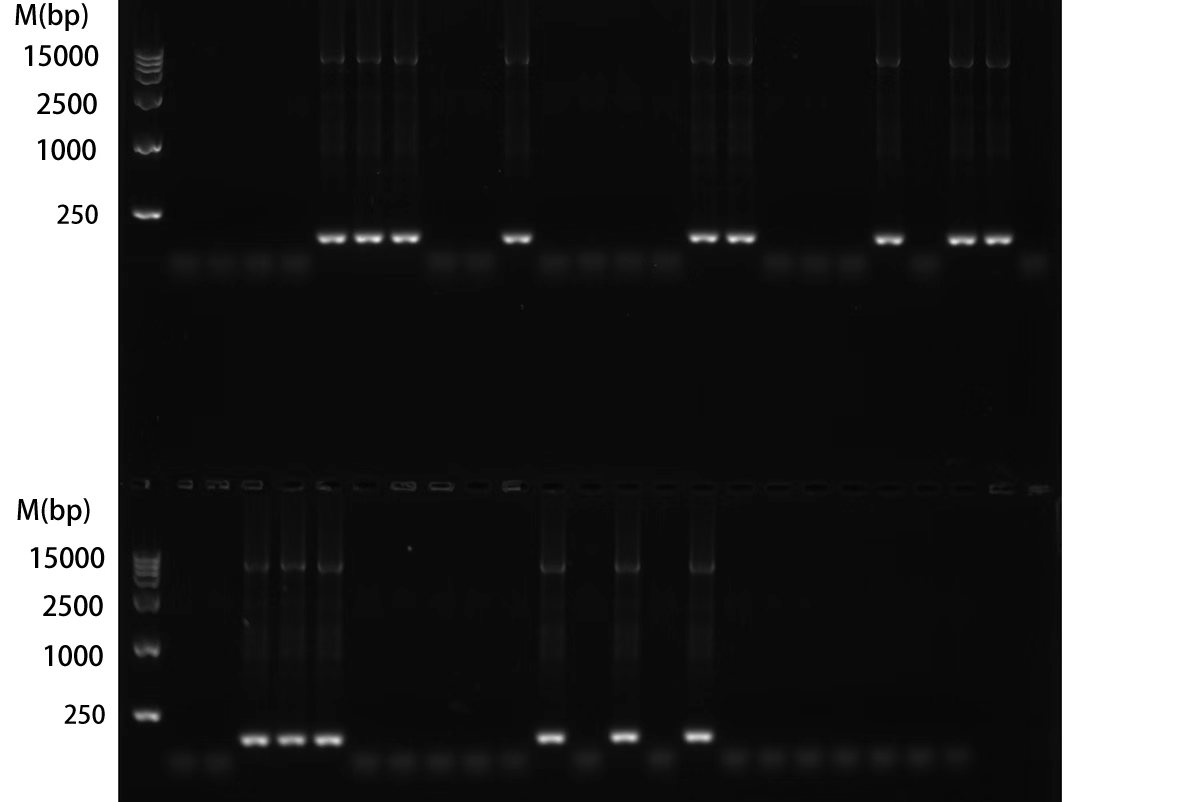

Supplement: Supplementary Figure S2 — PCR screening results for Corcyclo-2 in cormorant fecal samples. [file Image_2.jpeg]

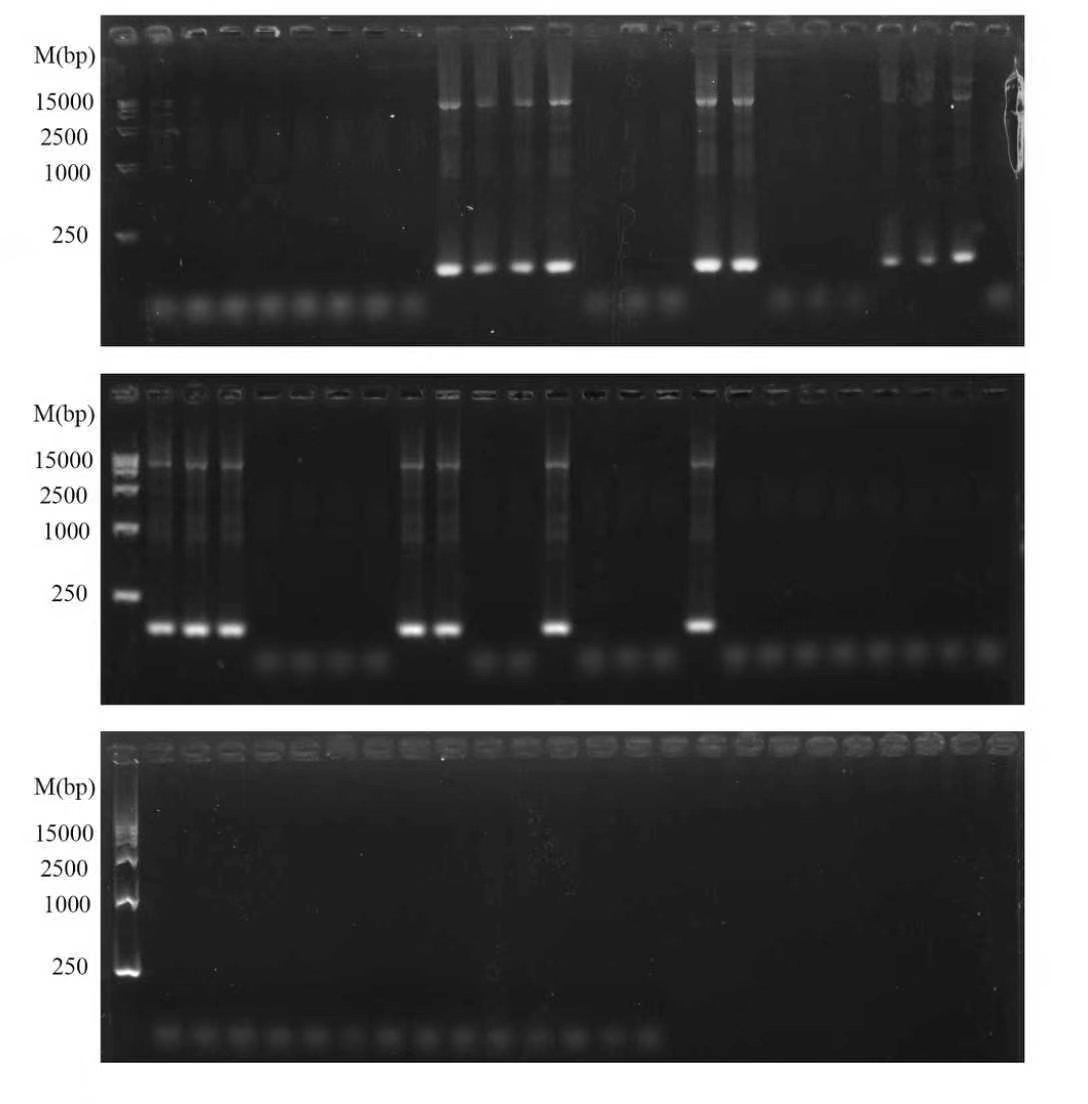

Supplement: Supplementary Figure S3 — PCR screening results for Corcyclo-2 in chicken fecal samples. [file Image_3.jpeg]

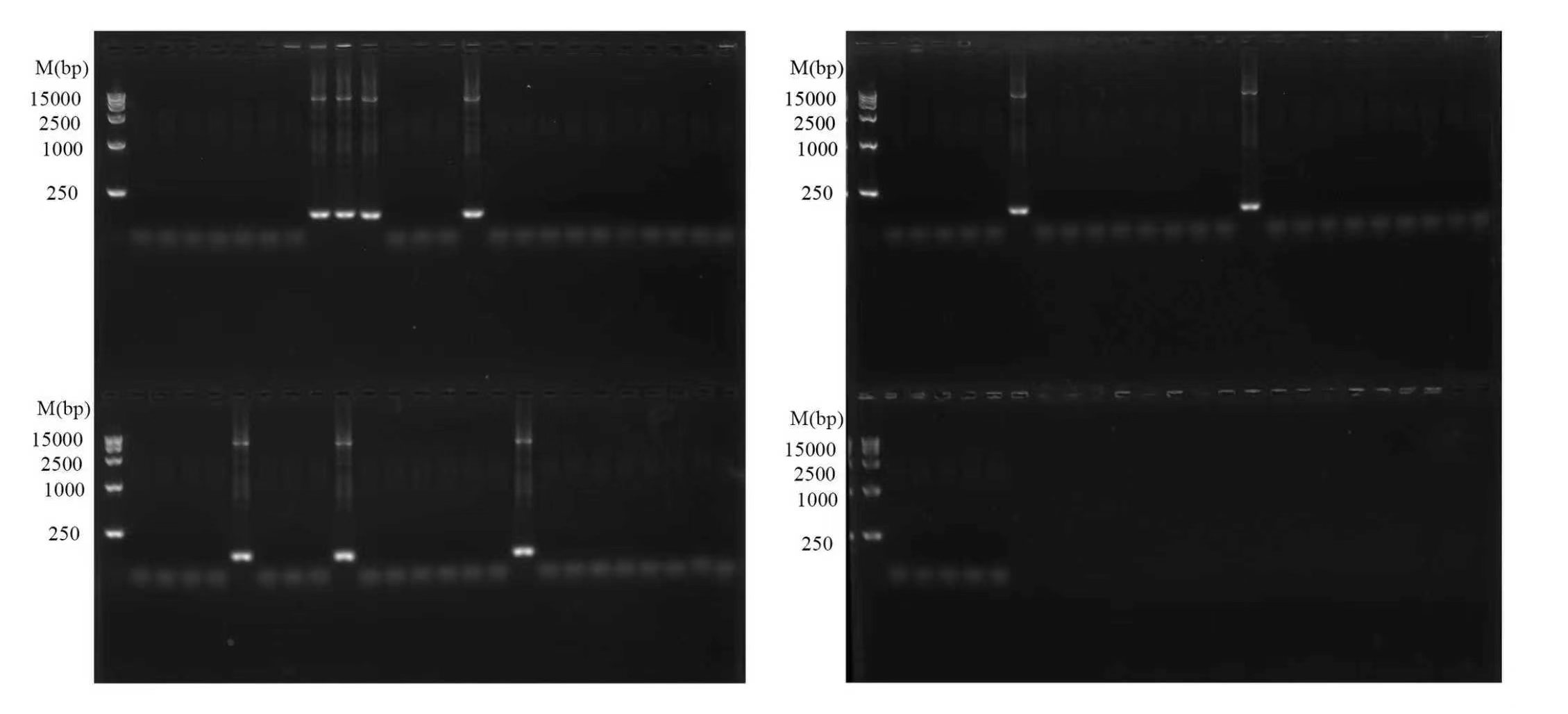

Supplement: Supplementary Figure S4 — PCR screening results for Corcyclo-2 in duck fecal samples. [file Image_4.jpeg]
